# Analysis of BAC end sequences in oak, a keystone forest tree species, providing insight into the composition of its genome

**DOI:** 10.1186/1471-2164-12-292

**Published:** 2011-06-06

**Authors:** Patricia Faivre Rampant, Isabelle Lesur, Clément Boussardon, Frédérique Bitton, Marie-Laure Martin-Magniette, Catherine Bodénès, Grégoire Le Provost, Hélène Bergès, Sylvia Fluch, Antoine Kremer, Christophe Plomion

**Affiliations:** 1 INRA, URGV, Plant Genomics Research, F-91057 Evry, France; 2 INRA, UMR1202 BIOGECO, F-33610 Cestas, France; 3 INRA, CNRGV, F-31326 Castanet, France; 4 Austrian Institute of Technology, A-2444 Seibersdorf, Austria

## Abstract

**Background:**

One of the key goals of oak genomics research is to identify genes of adaptive significance. This information may help to improve the conservation of adaptive genetic variation and the management of forests to increase their health and productivity. Deep-coverage large-insert genomic libraries are a crucial tool for attaining this objective. We report herein the construction of a BAC library for *Quercus robur*, its characterization and an analysis of BAC end sequences.

**Results:**

The *Eco*RI library generated consisted of 92,160 clones, 7% of which had no insert. Levels of chloroplast and mitochondrial contamination were below 3% and 1%, respectively. Mean clone insert size was estimated at 135 kb. The library represents 12 haploid genome equivalents and, the likelihood of finding a particular oak sequence of interest is greater than 99%. Genome coverage was confirmed by PCR screening of the library with 60 unique genetic loci sampled from the genetic linkage map. In total, about 20,000 high-quality BAC end sequences (BESs) were generated by sequencing 15,000 clones. Roughly 5.88% of the combined BAC end sequence length corresponded to known retroelements while *ab initio *repeat detection methods identified 41 additional repeats. Collectively, characterized and novel repeats account for roughly 8.94% of the genome. Further analysis of the BESs revealed 1,823 putative genes suggesting at least 29,340 genes in the oak genome. BESs were aligned with the genome sequences of *Arabidopsis thaliana*, *Vitis vinifera *and *Populus trichocarpa*. One putative collinear microsyntenic region encoding an alcohol acyl transferase protein was observed between oak and chromosome 2 of *V. vinifera.*

**Conclusions:**

This BAC library provides a new resource for genomic studies, including SSR marker development, physical mapping, comparative genomics and genome sequencing. BES analysis provided insight into the structure of the oak genome. These sequences will be used in the assembly of a future genome sequence for oak.

## Background

*Quercus *(oak) belongs to the Fagaceae family which also contains the genera *Castanea *(chestnut), *Fagus *(beech), *Lithocarpus *(stone oaks) and *Castanopsis*. Oaks constitute a major component of northern hemisphere forests, extending from temperate to tropical regions [1]. Oaks provide raw material for different uses but also afford important environmental services (carbon sequestration, energy production, water cycle etc.). These long-lived organisms are also considered good models for studies of the short- and long-term mechanisms of adaptation to the abiotic and biotic constraints associated with global climate change, because they grow under a wide range of soil and climatic conditions [1]. The traits involved in adaptation are complex, so exploration of the entire genome is required to locate the genes involved.

The species of the *Quercus *genus are diploid (2 n = 24). Haploid DNA content varies between the species, ranging from 539 Mb in *Q. velutina *to 921 Mb in *Q. coccifera *and *Q. ilex*, and 740 Mb in* Q. robur *[2], corresponding to five times the size of the *Arabidopsis *genome (using the estimate of 157 Mb from Bennett *et al. *2003 [3]) and approximately twice the size of the poplar genome (using the estimate of 485 Mb from Tuskan *et al. *2006 [4]).

Large collections of oak expressed sequence tags (ESTs) have been generated from various tissues and developmental stages, including 130,000 Sanger sequences and 2 M 454-reads, available from public databases [5]. This catalog constitutes a useful resource for detecting candidate genes controlling traits of interest and for the development of new genetic markers for forward genetics approaches (linkage mapping and QTL detection, association mapping) for dissection of the genetic architecture of adaptive traits [6-9]. However, little is known about the overall structure of the oak genome.

Bacterial artificial chromosome (BAC) genomic libraries provide a source of large genomic DNA insert clones for physical mapping, gene isolation, comparative studies of gene organization between species and sequencing projects [10,11]. Despite carrying large inserts of genomic DNA (up to 200 kb), BAC clones display low rates of *de novo *rearrangement and are easy to handle. BAC libraries are thus widely used as genomic tools for diverse organisms, including forest tree species (Additional file 1). With the recently introduced strategies of genome sequencing combining BAC end Sanger sequences (BESs) with sequence reads from next-generation sequencing technologies, it has now become possible to sequence the oak genome. In this context, the use of BESs should make it possible to develop scaffolding over long distances, thus ensuring the long-range contiguity of the assembly particularly for large and heterozygous genomes [12,13]. We had two main aims in this study: i) to construct and characterize a BAC library for *Quercus robur*, and ii) to characterize the composition of the oak genome by sequencing and analyzing BESs. A 12 × coverage library was obtained and an analysis of 20,056 BESs provided insight into the structure and composition of the oak genome.

## Results and Discussion

### BAC library characterization

#### Estimation of mean insert size

**Figure 1:**
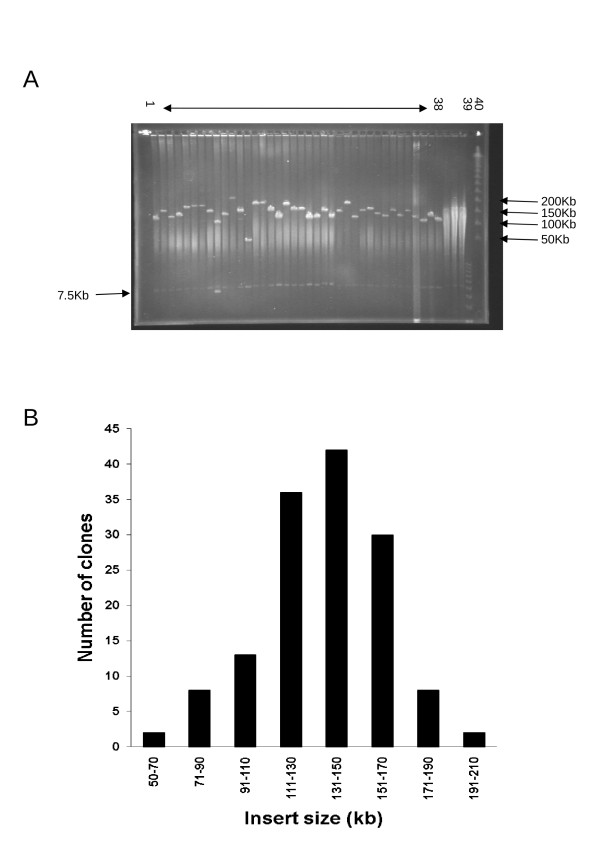
**Estimation of mean insert size in the oak BAC library**. A - Pulsed-field gel electrophoresis of 38 BAC clones DNA digested with *Not*I (Lanes 1-38) The 7.5 kb band is from the cloning vector. Lane 39 molecular marker 1 kb+ Invitrogen, Lane 40 size standard PFGE marker (Lambda Ladder PFG marker New England Biolabs). B - Insert size distribution of clones from the *Quercus robur *BAC library. The x-axis shows the size of the insert in kb. The y-axis indicates the number of clones.

This library consists of 92,160 clones stored into 240 384-well plates. We evaluated the mean size of BAC inserts by randomly selecting 189 clones, extracting their DNA and digesting it with the rare cutter enzyme *Not*I for analysis by PFGE (Figure 1A). The mean size of the inserts was 135 kb with insert size ranging from 50 kb to 205 kb. Over 85% of the BAC clones carried an insert larger than 90 kb and only 1% had inserts smaller than 50 kb (Figure 1B). The percentage of empty clones was estimated at 7% for the total library (Table 1). The empty clones probably resulted from problems in colony picking.

**Table 1:** Characteristics of the oak BAC library

Cloning vector	Pindigo BAC 536
Partial digest enzyme	*Eco*RI
Number of clones	92160
Number of 384-well plates	240
Missed wells	~7%
Mean insert size	135 kb
Minimum insert size	50 kb
Maximum insert size	205 kb
Chloroplast contamination	2.20%
Mitochondrial contamination	-
Number of genome equivalents	12×

#### Screening the library for cytoplasmic DNA sequences

We investigated the frequency of BAC clones containing chloroplast (cp) and mitochondrial (mt) DNA sequences in the library by carrying out PCR with specific primers to screen a subset of the library consisting of 984 individual BAC clones. Amplification products were detected for 22 BAC clones, indicating a low frequency of clones derived from the chloroplast genome (2.2%). No BAC clone containing mt DNA was detected (Table 1).

#### Estimation of genome coverage

The approximate haploid genome size of *Quercus robur *has been estimated at 740 Mb [2]. Based on mean insert size, the frequency of cytoplasmic sequences and the number of empty clones, the coverage of this library was estimated at 12×. We used the Clarke - Carbon equation [14] to estimate the probability of covering the genome: N = ln(1-P)/ln(1-[I/GS]), where N is the number of clones in the library, GS is genome size, and I is the insert size. In our case, the probability of recovering any sequence of interest from the library was more than 99%. Moreover, the high degree of genome coverage and the mean insert size of 135 kb make this library suitable for diverse applications such as physical mapping, map-based cloning and genome sequencing.

#### Depth of genome coverage

The theoretical genome coverage of the BAC library was validated by PCR screening of the library with 60 genetic markers detecting unique loci (5 per linkage group). Library screening was facilitated by forming plate pools for 127 plates corresponding to the equivalent of seven genomes. For a unique co-dominant locus, we expected a mean of seven hits. All but three of the markers detected at least one positive pool plate. In total, 430 pool plates were identified and the number of BAC clones detected by each marker ranged from 1 to 20, giving a mean of seven BAC clones per marker. Thus, the calculated depth of coverage was confirmed by screening the library with 60 genetic markers by PCR (Additional file 2). However, the library is not entirely random because not all the sequences tested were represented. This bias may be due to the use of *Eco*RI for cloning or may reflect the presence of genomic regions in which the *Eco*RI site is underrepresented. The use of several enzymes is usually recommended to achieve complete representation of the genome [15]. We therefore constructed a second BAC library for the same *Q. robur *genotype, using *Hin*dIII as the cutting enzyme (results not shown). Both libraries are available at the CNRGV [16] and PICME [17] repository centers for library and clone distribution. A set of 15,000 clones is also being sequenced (both ends) to characterize this second library.

### BAC end sequences

We sequenced 14,976 BAC clones from both ends. After trimming of the Sanger reads for vector, *E. coli *contamination and low read quality, we retained 20,056 (66.96%) BAC ends for further analysis [GenBank: HN154083 - HN174138]. We had forward and reverse sequences for 71% of these BESs (7,131), giving 7,131 BES mate pairs. The mean length of high-quality reads was 599 bp with a mean GC content of 35.33% (Table 2). Although lower than the GC content estimated by colorimetry (39.9% [18]), this figure is similar to that found for the complete genomes of *Arabidopsis *(36% - [19]), poplar [4], yellow poplar [20] (34%) and grapevine (35% - [21]). However, GC content may be biased by the restriction enzyme used to generate the BAC clones, as found in tomato [22]. A noticeable difference in GC content was observed between BESs with (36.71%) and without a protein signature (32.16%).

**Table 2:** Summary of BAC end sequencing

No. of good-quality BAC end sequences	20,056
Total base count	12,018,238
Minimum length	100 bp
Maximum length	967 bp
Mean length	599 bp
GC content	35.33%
*Chloroplast matches*	
Oak	2.60%
Poplar	1.20%
Grapevine	1.20%
*Mitochondrion matches*	
Grapevine	0.60%

Comparison of the BESs with the chloroplast (cp) genomes of oak (kindly provided by GG. Vendramin), poplar [GenBank: DQ424856] and grapevine [GenBank: EF489041] confirmed the low frequency of cp sequences in the library (<2%). The mitochondrial (mt) genome of oak has not yet been sequenced, so we searched for homologous mt sequences by comparison with the grapevine mt genome. Less than 1% of our BESs showed significant matches with the grapevine mt genome [GenBank: NC_012119]. These values are consistent with the estimates obtained by PCR screening with cp- and mt-specific primers.

### Classical repeat analysis and classification

Based on similarity searches in the repeat database, 5.88% of the nucleotides in the oak BESs were identified as belonging to known repeats. Class I retrotransposons were the most abundant repeats, with a total of 2,196 retroelements (5.51% of the BESs). BESs homologous to retrotransposons were further classified as LINE (0.65%) or LTR elements (4.86%) (Ty1/copya, 61.50%; Gypsy/DirS1, 37.33%) (Table 3). Ty1/copia elements were the most abundant retroelements. Similar figures have been reported for the apple [23], grapevine [24], carrot [25] and banana [26] genomes. By contrast, gypsy retroelements are the most abundant in clementine, poplar, *Arabidopsis *and rice [27]. The proportion of retrotransposons was half that reported for rapeseed (12.3% - [28]), *Arabidopsis *(10% - [19]) and black cottonwood (12.6% - [4]) and was much lower than that for carrot (22.6% - [25]) and grapevine (38.8% - [20]). However, the low repeat content may be due to the use of *Eco*RI in construction of the oak library. In tomato, *Eco*RI BESs were found to contain far fewer repeats than *Hba*I or *Mbo*I BESs. In potato, *Eco*RI BESs also had lower retroelement content than *Hin*dIII BESs. *Eco*RI shows methyl sensitivity limiting the restriction of highly methylated regions of the genome where repeat elements are usually found.

**Table 3:** Classification and distribution of known plant repeats in the BAC end sequences

Class	Number of elements	% of nucleotides	Length (bp)
**Retroelements**	**2196**	**5.51**	**662,150**
*LINEs:*	*318*	*0.65*	*78,495*
RTE/Bov-B	18	0.04	4,649
L1/CIN4	297	0.61	73,545
*LTR elements:*	*1,878*	*4.86*	*583,655*
Ty1/Copia	1,155	3.03	364,184
Gypsy/DIRS1	701	1.8	216,428
**DNA transposons**	**206**	**0.37**	**43,907**
**Total interspersed repeats**	-	**5.88**	**706,119**
**Small RNA**	**54**	**0.1**	**12,218**

a - Numbers indicate the percentages of BESs displaying similarity to a repeat from the indicated category.

### Identification of novel repeats

**Figure 2:**
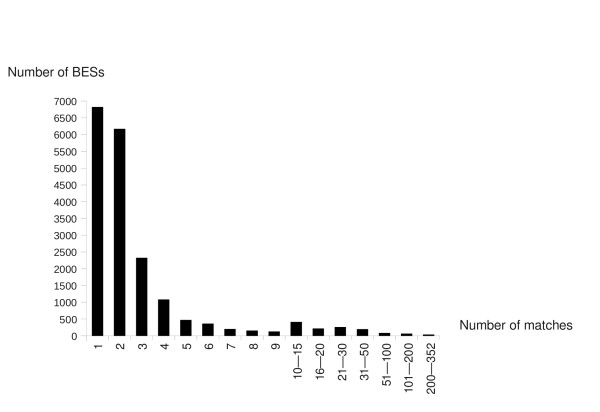
**Estimation of redundancy within oak BESs**. Distribution of the number of masked BESs with at least one significant alignment with another BES in the dataset. BESs were masked for repetitive DNA and low-complexity sequences identified with RepeatMasker software, using the Viridiplantae section of the RepBase database. Values on the y-axis represent the number of BESs matching the number of BESs listed on the x-axis (between one and 352 BESs).

Similarity-based repeat detection may be limited by the size and diversity of the repeat database. We therefore carried out a self-comparison of the BESs, to identify previously unknown putative repetitive sequences. If a region of a BES has multiple hits with many other BESs, these sequences probably correspond to novel repetitive sequences. Even with the stringent threshold requirement -- that each 100 bp window matches a BES with at least 90% identity -- 62.9% (12,595) of the oak BESs matched at least one other BES (Figure 2). Similar results were obtained when repetitive elements and low-complexity sequences were masked, slightly decreasing the number of matching BESs from 12,595 to 12,138 (*i.e. *2.4% decrease). For the purposes of comparison, we performed the same analysis on two fruit trees and one forest tree: *Carita papaya *(40,489 BESs), *Citrus clementina *BESs (45,839 BESs clementine genome) and *Populus trichocarpa *(13,249 BESs [GenBank: HN280500 - HN291979]. We found that 63.8%, 74.57% and 72% of the papaya, clementine and poplar BESs respectively matched at least one other BES. If we masked known repetitive elements and low-complexity sequences, 62.95%, 72.95% and 47.8% of BESs, respectively, still matched at least one other BES. As for oak, masking papaya, clementine BESs for known repeat elements only slightly decreased redundancy in the BESs. However, the number of residual redundancies in oak, papaya and clementine BESs was greater than in poplar. In order to estimate the number of matches for which BES could be classify as repeat, we consider the question as a hypothesis test. We determine the threshold T for which the Type I Error is lower or equal to a fix alpha = 5% for a null hypothesis « the sequence is not repeated » and an alternative hypothesis « the sequence is repeated i.e. the number of match with other BES is greater or equal to T». If the oak genome was composed totally of random nucleotides (i.e. the nucleotides are independent and the frequency of each one is ¼), then the probability that two 100 bp sequences with 90% sequence identity have a match equals p0 = 0.2590 = 6.5 × 1.e-55. Under the null hypothesis, the distribution of the number of match is a binomial distribution with 19,999 trials and a probability of success equals to p0. Since the probability p0 is close to 0, the probability to have no match equals 1. That means that as soon as a BES has a match with another BES it can be considered repeated. If the calculation of p0 is done from the empirical frequencies observed on the oak genome, p0 = 7.6 × 1e-52 but the conclusions are the same since the probability to have no match equals 1. That is to say that as soon as a BES has a match with another BES, it can be considered repeated. This result suggests that oak BESs contained other repeat elements not yet identified in other plants.

### Characterization of oak repeat elements (ORE)

Despite the masking of known repeat elements in our BESs, 60.5% could be considered as putative repeats. Datema *et al. *carried out a similar analysis on potato and tomato [22]. Based on the criterion that at least 50% of a given sequence matches another BES with at least 90% identity, 52% of the nucleotides in the tomato BESs displayed matches with at least one other tomato BES and 19% displayed matches with at least five other BESs. Potato BESs displayed a lower degree of redundancy than those of tomato; 39% of the nucleotides in the potato BESs had a hit with at least one other BES, and 12.9% had a hit with at least five other BESs. The authors concluded that the remaining redundancy after repeat masking might correspond to novel repetitive or duplicated sequences. In carrot, high levels of redundancy were found to be due to repetitive elements not previously identified in other plants [25]. By considering the BES with a minimum of 6 hits, the authors characterized 11 carrot repetitive elements. In the oak BES data set we identified 93 repeat sequences among the 2,948 BESs presenting at least six matches with other BESs. For confirmation that these sequences were unique to the oak genome, we queried them against the NCBI GenBank non-redundant nucleic acid sequence database, the NCBI GenBank EST database (excluding oak ESTs), the Swissprot database, the TIGR Plant Repeat Databases, the Triticeae repetitive sequence database and the GIRI repeat database. None of these repeat sequences matched protein sequences in the Swissprot database but 52 repeat sequences matched at least one accession in the other databases. These sequences were removed from our list of putative oak repetitive elements (OREs). Of the remaining 41 OREs, 19 matched oak ESTs, 1 motif matched Fagaceae ESTs (*Quercus and Castanea*), 1 motif matched a *Quercus suber *retrotransposon 'Qsub2' in the NR database, and 20 motifs specifically matched oak BESs corresponding to unknown repetitive sequences (Additional file 3). These 41 OREs were present in seven to 119 copies in the BES database and their sizes ranged from 80 bp to 224 bp (Additional file 4). Overall, these OREs matched 1,459 BESs, covering 151,565 bp and accounting for almost 1.26% of the total BES length. Extrapolating to the level of the oak genome, there could be as many as 7,327 copies of the most frequent ORE. Similarly, four other OREs may be present more than 4,000 times. Thus, in addition to the repetitive DNA fraction identified by classical analysis (5.88% - Table 1), the 41 OREs and 52 repeat sequences bring the total repetitive DNA content to a minimum of 8.94%.

### Simple sequence repeats (SSRs)

**Figure 3:**
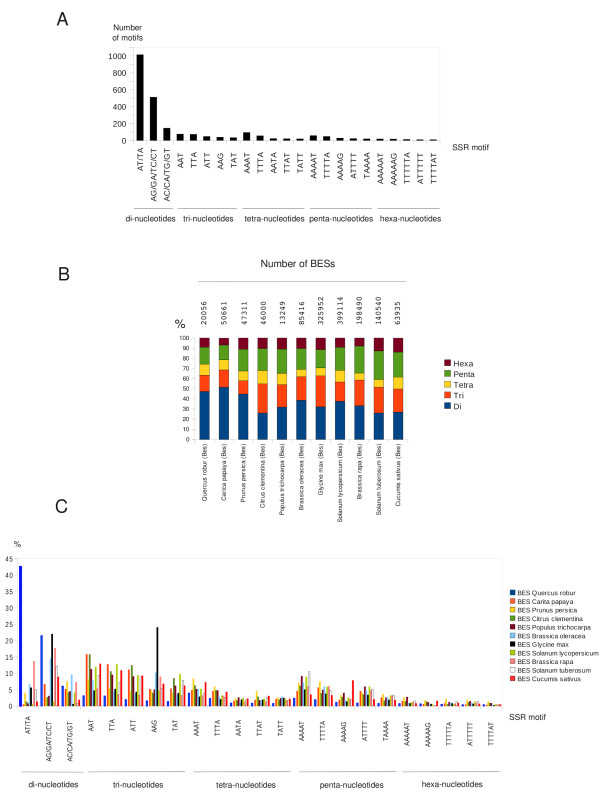
**Distribution of SSR motifs**. A - Distribution of the most abundant SSR motifs in oak BESs. The values on the y-axis indicate the fraction of SSRs displaying the motifs listed on the x-axis. SSR motifs were identified with MREPS 2.5. All the dinucleotide motifs are represented. Only the five most abundant tri-, tetra-, penta- and hexanucleotide motifs are listed. B - Distribution of di-, tri-, tetra-, penta- and hexanucleotide motifs identified by MREPS, using the same parameters in 10 BAC end sequences libraries published in the NCBI dbGSS database, normalized for cross-species comparisons. C- Distribution of the most abundant SSR motifs found in oak BES in 10 other BES datasets: *Carita papaya *(50,661 BESs), *Prunus persica *(47,311 BESs), *Citrus clementina *(46,000 BESs), *Populus trichocarpa *(13,249 BESs), *Brassica oleracea *(85,416 BESs), *Glycine max *(325,952 BESs), *Solanum lycopersicum *(399,114 BESs), *Brassica rapa *(198,490 BESs), *Solanum tuberosum *(140,540 BESs), *Cucumis sativus *(63,935 BESs). The values on the y-axis indicate the fraction of SSRs displaying the motifs listed on the x-axis. SSR motifs were identified with MREPS 2.5, using the same parameters as for oak BESs. The values have been normalized for cross-species comparisons.

In total, 3,531 SSRs with a motif length of between two and six nucleotides were detected among the oak BESs corresponding to one SSR per 3.45 kb (29 SSRs per 100 kb) of genomic sequence. This frequency was found to be higher than in other plant species (Additional file 5). Dinucleotide motifs were the most abundant (1,672 SSRs, 47.35%), followed by penta- (590 SSRs, 16.71%), tri- (564 SSRs, 15.97%), tetra- (386 SSRs, 10.93%) and hexa-nucleotide motifs (319 SSRs, 9.03%) (Figure 3A). The most abundant dinucleotide SSR motifs in oak BESs were AT/TA (60.71%) and AG/GA/TC/CT (30.62%) (Figure 3A). No GC motifs were found. Dinucleotide motifs were also the most abundant motifs in other species, such as *Carita papaya *(51.47%) and *Prunus persica *(44.72%), followed by penta- and tri-nucleotide motifs (14.53% and 17.01%, respectively, for *C. papaya *and 21.41% and 13.17% for *Prunus persica*) and, finally tetra- and hexa-nucleotide motifs. Conversely, for other species, approximately equal proportions of di-, tri- and pentanucleotide motifs were found (Figure 3B and Additional file 5). In addition, the SSR motif content of oak was found to be significantly different from that of other species (Figure 3C). In the oak Unigene dataset [5], di- and trinucleotide motifs were the most frequent (36.25% and 36.63%, respectively) followed by tetra- (10.45%) and hexanucleotide motifs (9.90%). Trinucleotide SSRs (mainly AAG) were twice as frequent as in the Unigene set. The enrichment of trinucleotide SSRs in ESTs is consistent with previous reports of SSR abundance in the gene space (discussed in [5] and [9]).

### Gene content

Once repeats were masked, 2,712 BESs (13.5% of total BESs) were found to match at least one *A. thaliana *sequence in the NR database. We found that 0.33% and 0.11% of these 2,712 BESs were homologous to cp and mt sequences, respectively. A total of 1,823 masked BESs (9.1% of the BESs) matched at least one *A. thaliana *sequence in the Swissprot database (25,056 significant alignments) (Additional file 6), 166 (0.83%) and 66 (0.33%) of which matched a chloroplast- or mitochondrion-encoded protein sequence, respectively. The number of cp hits was in the range of chloroplast contamination estimated by PCR (*i.e *2.2% - Table 1). We found that 1,461 BESs matched an *A. thaliana *sequence in both the NR and Swissprot databases, including 0.55% (8 BESs) of cp and 0.14% (2 BESs) of mt sequences. We found that 5,250 masked BESs (26.18%) matched at least one oak EST sequence in the Oak Unigene dataset (15,359 significant alignments), and among these sequences, we identified 4.21% of cp and 0.1% of mt protein-coding sequences. Among these 5,250 BESs, 2,018 (38.44%) also matched at least one sequence in Swissprot, NR or both databases (Additional file 7).

Based on the number of BESs matching at least one *A. thaliana *sequence in the Swissprot database (1,591), the mean sequence length of the BES (599 bp), the size of the oak genome (740 Mb), the total size of the BESs (9,535 kb) and the mean size of a gene (2 kb - [19]), we estimated a number of 29,340 genes. Bioinformatics' analysis on oak unigene set revealed that 11% of them have no homology with genes in Arabidopsis [5], taking into account this result we estimated the gene content of the whole genome of at least 32467 genes. This estimated number of genes is consistent with the gene number for a fully sequenced plant genome.

### Functional annotation

**Figure 4:**
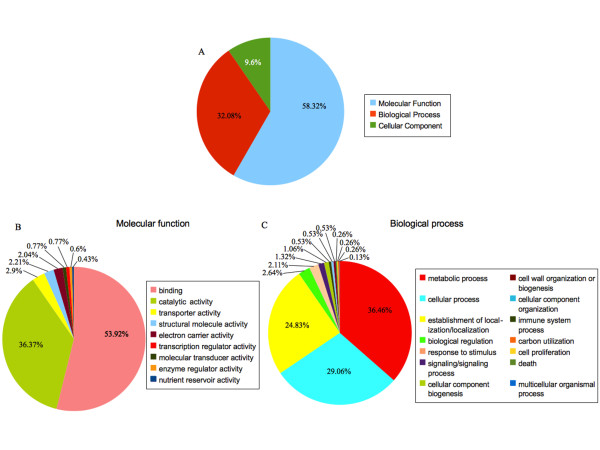
**Gene ontology classification of the 1,823 oak BESs significantly aligned with *A. thaliana *sequences in the Swissprot database**. A - Masked BESs were annotated as related to Molecular Function, Biological Process and Cellular Component categories. B - Molecular Function annotation of the BESs grouped into 9 higher level terms of the Gene Ontology. C - Biological Process annotation of the BESs grouped into 12 higher level terms of the Gene Ontology.

Among the 1,823 oak BESs significantly aligned with *A. thaliana *sequences in Swissprot, 799 BESs were associated with at least one GO term (Additional file 8). A total of 261 GO terms were assigned to these 799 oak BESs on the basis of matches in the Pfam database: 492 BESs were annotated with at least one of the 95 terms of the Biological Process category, 753 were annotated with at least one of the 136 terms of the Molecular Function category and 208 were annotated with at least one of the 30 terms of the Cellular Component category (Figure 4A). Most terms occurred at relatively low frequency. Only 38 of the 261 GO terms assigned to the BESs occurred ten or more times in this dataset. A large proportion of the 1,171 assignments to the Molecular Function category were associated with the Binding (53.92%) and Catalytic Activity (36.37%) categories (Figure 4B). Most of the 633 assignments to the Biological Process category concerned the Metabolic Process (36.46%), Cellular Process (29.06%) and Localization (24.83%) categories (Figure 4C).

**Figure 5:**
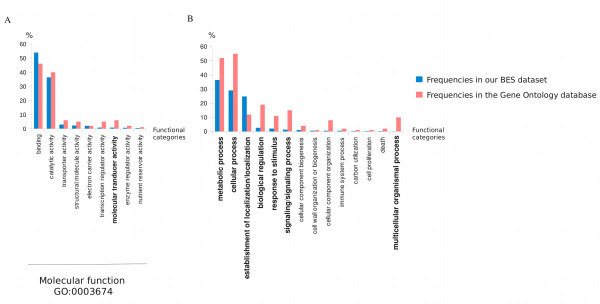
**Comparison of the distribution of functional annotations of oak BESs with the functional annotations in the Gene Ontology database**. Distribution of Gene Ontology functional annotations in the oak BES dataset (blue) and in the Gene Ontology database (pink) belonging to A - the Molecular Function category (GO:0003674) and B - the Biological Process category (GO:0008150). We identified the functional categories (**in bold**) with frequencies in our BES dataset different from those in the Gene Ontology dataset, through chi^2 ^tests (P < 0.05).

Within these two categories -- Molecular function (Figure 5A) and Biological process (Figure 5B) -- the distribution of the functional annotations of our BESs differed significantly from the global Gene Ontology database. Indeed, a chi^2 ^test (P < 0.05) showed that the Metabolic Process, Cellular Process, Biological Regulation, Response to Stimulus, Signaling and Molecular Transducer activity categories were significantly underrepresented in our dataset. By contrast, the Localization category was twice as frequent.

### Comparative genome mapping

We found that 176 of the 20,056 oak BESs that were compared with the *V. vinifera *genome presented at least one match. These matches were divided into seven categories, as shown in the last seven columns of Additional file 9. The **'single end' **category corresponds to BAC end pairs for which only one of the two sequences matched a sequence in the *V. vinifera *genome Most of the matches (415) were of this type. Twenty BES pairs for which BESs from the same BAC matched the *V. vinifera *genome (not necessarily the same chromosome) were assigned to the **'paired-end' **category. The **'colocalized' **category contained eight BAC end pairs that matched the same *V. vinifera *chromosome. The distance between the paired matches for seven of these eight BES pairs was either smaller than 15 kb or larger than 250 kb (**'gapped' **category). For one of the eight BES pairs, 20 hits were detected with the *V. vinifera *genome and all of these intertwined alignments fell into the **'no-gapped' **category for chromosome 2 of *V. vinifera*. The last two categories corresponded to BACs for which both end sequences matched the genome, at points 15 to 250 kb apart on the *V. vinifera *and *P. trichocarpa *genome, either in the correct orientation with respect to each other (**'collinear'**) or rearranged with respect to each other (**'rearranged'**). One of the eight BES pairs matching the same *V. vinifera *chromosome fell into the 'collinear' category, suggesting the presence of one putative microsyntenic region between oak and chromosome 2 of *V. vinifera*. This region contains the *GSVIVG01022745001 *gene [29], which encodes an alcohol acyl transferase protein very similar to that encoded by the *Lupinus albus Q5H873_LUPAL *gene and involved in competition with other plant species and in the synthesis of defense compounds active against pathogenic organisms [30]. The sequence of the protein encoded by *GSVIVG01022745001 *matched 88 sequences in the Oak Unigene set [5], all classified as having GO:0016747 Transferase activity, transferring acyl groups other than amino-acyl groups in the Gene Ontology classification.

Only three pairs of the BESs mapped to the *P. trichocarpa *genome (Table 4 and Additional file 10). For two of these pairs of BESs, both BESs matched the same chromosome. However, none of the oak BESs matched to points in the *P. trichocarpa *genome within 15 to 250 kb of each other.

**Table 4:** BlastN hits between oak BESs and the *Vitis vinifera*, *Populus trichocarpa *and *Arabidopsis thaliana *genomes

	Hit	No. hits (BESs)	Single end (BESs)	**Paired-end (pairs**^**1**^**)**	Co-localized (pairs)	Gapped (pairs)	Non-gapped (pairs)	Collinear (pairs)	Rearranged (pairs)
*V. vinifera*	176 BESs 1050 alig^2^.	19880	136	20	8	7	1	1	0
*P. trichocarpa*	81 BESs 184 alig.	19975	75	3	2	2	0	0	0
*A. thaliana*	134 BESs 334 alig.	19922	102	16	16	0	16	8	8

^1 ^pair = BES pair

^2 ^alig. = significant alignment

We repeated this analysis for the *A. thaliana *genome. For the 16 BES pairs identified as 'co-localized', both ends matched to the chloroplast molecule *(i.e*. contamination 0.2%) (Table 4).

In similar investigations in the *A. thaliana *genome, Datema *et al. *[22], identified very few regions of microsynteny in potato (one collinear and one rearranged sequence) and tomato (three collinear and one rearranged). Tomato displayed a higher degree of synteny with *P. trichocarpa*, with 51 collinear sequences and 22 rearranged sequences.

## Conclusions

We constructed the first genomic BAC library for the genus *Quercus*. It was built for a genotype involved in controlled crosses for genetic mapping and QTL detection. The estimated genome coverage of 12 × was confirmed by PCR screening of 60 genetic markers evenly distributed over the genetic linkage map. Both genome coverage and the mean insert size of 135 kb make this library useful for physical mapping and map-based cloning approaches for adaptive trait QTLs and genome sequencing. We carried out a preliminary examination of the composition of the genome sequence by generating 20,056 BESs and searching for sequence similarities. The sequences contained a relatively small proportion of the known repetitive DNA sequences (5.88%). However, 3.06% of the BESs constituted new repeat sequences. Protein-coding regions accounted for 13.5% of the BESs. Only 176 and 81 matches were found between oak and grapevine or oak and poplar respectively, suggesting that studies of the oak genome will provide new insight into the organization and function of plant genomes.

## Methods

### Plant material

The *Quercus robur *genotype named 3P was selected for BAC library construction. It was used as the female parent of an intraspecific control cross, 3P × A4 [31]. A dense genetic map is available [9] and QTL for adaptive traits have already been described for this genotype [6,7,32]. Young leaves were collected from an adult tree and incubated 3 days in the dark at 4°C. The leaves were washed in double-distilled H_2_O and frozen in liquid nitrogen, then stored at -80°C until use.

### BAC library construction

The BAC library was constructed at the Clemson University Genomic Institute (CUGI, http://www.genome.clemson.edu/services/bacrc/BAC_library). Briefly, high-molecular weight DNA was partially digested with *Eco*RI and subjected to size selection via pulsed-field gel electrophoresis. Size-selected DNA was ligated into the vector, pBeloBAC536. *E. coli *strain DH10B was electroporated with the ligation products. Recombinant white colonies were arrayed as individual clones in 240 384-well microtiter plates containing Freezing Medium (FM) (13 mM KH_2_PO_4_, 36 mM K_2_HPO_4_, 1.7 mM sodium citrate, 6.8 m (NH_4_)_2_SO_4_, 4.4% v/v glycerol) with 12.5 μgml-1 chloramphenicol.

### BAC clone characterization/BAC insert sizing

BAC DNA was prepared by a standard alkaline lysis method [33], from 3 ml of overnight culture in 2YT supplemented with 12.5 μg/ml chloramphenicol. The pellet was resuspended in 40 μl of TE (10:1). We estimated mean insert size and determined the distribution of clone sizes, by digesting 10 μl of BAC DNA miniprep with 10 U of *Not*I enzyme. Digested BAC DNA was fractionated by PFGE (CHEF-DRIII, Biorad, USA) in a 0.5% agarose gel in 0.5 × TBE buffer (0.09 M Tris-borate, 0.09 M boric acid, 0.002 M EDTA), with a 1-40 s linear ramp, 6 V/cm, 14°C and a 13 h run time. The gel was then stained with ethidium bromide and photographed with a Gel Doc apparatus (Bio-Rad, Hercules, California). The size of the insert in each BAC clone was determined by comparison with PFGE size standard markers (Lambda Ladder PFG Markers New England Biolabs, Ipswich, MA, USA).

### PCR screening for organelle contamination

Universal chloroplast primers CCMP2 (F-GATCCCGGACGTAATCCTG/R-ATGGTACCGAGGGTTCGAAT) and udt 5 (F-TAAATCTGGAAATCTGGGAA/R-TTGATACATAGACTTGCCAA) were used to estimate the level of chloroplast contamination, in individual tests of 984 BAC clones [34,35]. PCR was carried out on bacterial suspensions in 384-well plates. Each reaction was carried out in a 10 μl reaction volume containing 5 μM of each dNTP (Applied Biosystem, Carlsbad, CA, USA), 0.5 U *Taq *DNA polymerase (Applied Biosystems), 5 μM of each primer, 1 μl of 10 × PCR buffer, 50 μM MgCl2 (Applied Biosystems) and 20%(v/v) loading buffer [60% (w/v) sucrose, 5 mM Cresol Red in water]. Amplifications were performed with a GeneAmp 9700 PCR system (Applied Biosystems) programmed as follows: 94°C for 5 min, followed by 30 cycles of 94°C for 30 s, 55°C for 30 s, 72°C for 20 s, and then a final 5 min extension at 72°C. We used 3P genomic DNA as positive control. We then used the same procedure and mitochondrial primers F-GGTAATGGTTTGTTCCGATT/R-CATGCCTAGATACCCGAAGAC to evaluate mitochondrial DNA contamination of the library. PCR products were loaded onto 1% classical agarose gels in 1 × TAE buffer. Electrophoresis was performed at 300 mA for 30 min in 1 × TAE buffer. The gels were stained with ethidium bromide and photographed.

### PCR screening for SSR genetic markers

BAC clones from 127 384-well plates were replicated with a 384-well pin tool into microtiter plates containing 60 μl FM supplemented with 12.5-μg/ml chloramphenicol per well, and the plates were incubated overnight at 37°C. Each BAC clone was grown independently, to prevent growth-based competition. For each plate, we removed 20 μl from each well and added it to a single tube to create a plate pool. Dilutions of 1/20, 1/50 and 1/100 were tested for successful PCR amplification.

Sixty SSR markers (5 per linkage group from [9]) were used for BAC library screening, with 1:20-diluted plate pools as the DNA template. The PCR mixture was as follows: 2.5 μl of bacterial suspension was added to a 7.5 μl reaction mixture according to the procedure describe above. PCR was carried out with a touchdown program, as follows: initial denaturation for 5 min at 94°C, followed by 15 cycles of 20 s at 94°C, 20 s at a temperature of 65°C to 51°C with a decrease of 1°C at each cycle, 30 s at 72°C and a final 40 cycles of 20 s at 94°C, 20 s at 55°C and 30 s at 72°C. The program ended with a 5-minute step at 72°C. PCR products were separated onto agarose gels.

### BAC end sequencing

Thirty-nine plates were randomly selected for BAC end sequencing. This procedure was carried out with Applied Biosystems Big Dye Terminator chemistry and the results were analyzed on an ABI 3730 machine at the IG-CNS facility. Base calling was performed with PHRED [36]. Sequences were trimmed for vector and low-quality sequences with Seqtrim V0.110 [37].

### Identifying previously characterized repeats

Repeats in the oak BESs were identified by searches for similarity to sequences in the Viridiplantae section of the RepBase repeat database (release 05-10-2010) [38], with RepeatMasker 3.1.9 [39] and WU-blast [40]. Repeat density was then calculated as the percentage of nucleotides in the BESs with at least one hit matching the repeat database [41]. Repeat families were classified on the basis of annotation in the RepBase database.

### *Ab initio *Repeat identification

Oak BESs were first masked for known repeat elements with RepeatMasker. We then detected redundancy in the BESs with MegaBlast, by comparing the oak BESs with themselves (E-value = 10^-50^). Sequences with at least six hits were input into MEME V4.4.0 to identify DNA motifs (E-value = 10^-4^) [42]. We assessed the extent to which these motifs were unique, by using the resulting putative oak repeat elements (ORE) to query the NCBI GenBank non-redundant nucleic acid sequence database (Viridiplantae section - release 03-10-2010) [43], the NCBI GenBank EST database (Viridiplantae section - release 03-10-2010) [43] and the Oak Unigene set [5], with BlastN (E-value = 10^-5 ^for NR database and E-value = 10^-40 ^for EST databases).

We also used these sequences to query repeat databases including the TIGR Plant Repeat Databases (http://www.tigr.org/tdb/e2k1/plant.repeats/ - August 2010) [44], Triticeae repetitive sequence database (TREP) (http://wheat.pw.usda.gov/ITMI/Repeats/ - August 2010) [45], and GIRI repeat database (http://www.girinst.org/ - August 2010) [38], with BlastN and an E-value cut off of 10^-5^. Finally, we used the putative OREs as queries against the Swissprot database (release 2010-04) [46], with BlastX and an E-value cutoff of 10^-4^.

### Simple sequence repeats

Microsatellites were detected with Mreps 2.5 software [47]. Running parameters were set to return all SSRs with a motif length between 1 and 6 (*i.e*. mono-, di-, tri-, tetra-, penta- and hexanucleotide repeats). SSRs were at least 15 nucleotides long for tri- and pentanucleotide motifs, 16 nucleotides long for di- and tetranucleotide motifs and 18 nucleotides long for hexanucleotide motifs. The resolution parameter was set to 0, indicating that no irregular repetitive structure was allowed.

### Gene content

Gene content of the BESs was estimated through BLAST searches with Blastall 2.2.15. BESs were first masked for repeat sequences and low-complexity sequences with RepeatMasker 3.1.9 [39]. The BESs were then compared with the NCBI GenBank non-redundant protein database (*A. thaliana *- release 03-10-2010) [43], with BlastX [48]. We identified putative protein-coding regions, by comparing oak BESs with the Swissprot database (*Arabidopsis thaliana - *release 2010-04) [46], with BlastX. For all BlastX searches, an E-value cutoff of 10^-4 ^was used. In parallel, the gene content of the BESs was estimated against the Oak Unigene set, comprising 69,154 contigs and 153,517 singletons, by BlastN at a very high stringency (E value = 10^-50^) [5]. BlastN searches were performed with a minimum identity of 90% in each sliding window of 100 nucleotides. For each analysis, the percentage contamination with chloroplast and mitochondrial sequences was calculated.

### Functional annotation

Gene Ontology provides a system for classifying gene products according to three ontologies: Molecular Function, Cellular Component and Biological Process [49].

Oak BESs were functionally annotated by comparison with the HMMER 2.3.2 (Pfam V24.0) protein family databases, with InterProScan 4.6 [50,51]. GO terms from the Pfam annotations were extracted from the merged output file of InterProScan. For each GO term, the number of matching BESs was counted.

We performed the same analysis on Oak BESs significantly aligned with *A. thaliana *sequences in Swissprot.

### Comparative genome mapping

We tried to identify potential areas of microsynteny between oak and *Arabidopsis*, poplar or grapevine, by selecting paired BESs and mapping them onto the *Arabidopsis thaliana*, *Populus trichocarpa *and *Vitis vinifera *genome sequences with MegaBlast (Blastall 2.2.15) alignments. Whole-genome sequences from *A. thaliana*, *P. trichocarpa *and *V. vinifera *were downloaded from TAIR, Genoscope and URGI [52-54], respectively. The E-value cutoff was set at 10^-4 ^and BLAST hits were removed if they did not have a minimum identity of 90% in each sliding window of 100 nucleotides. A BAC was considered to display microsynteny to the target genome if both ends mapped to within 15 kb to 250 kb of each other. When the two ends were correctly oriented with respect to each other, the region was considered collinear. Otherwise, the region was considered to be rearranged between the two species. When a microsyntenic region was identified, we also compared the protein sequence with the Oak Unigene set [5], with tblastN. An E-value cutoff of 10^-5 ^was used.

## Authors' contributions

PFR coordinated the project and drafted the manuscript. FB performed the repeat analysis and characterized the library. CB^1 ^characterized the library. IL performed the bioinformatic analyses and drafted the manuscript with CP. MLMM performed the statistical analysis. GLP prepared and sampled the plant material. CB^2 ^was involved in the PCR screening of SSR markers. HB and SF were responsible for the storage of the two BAC libraries. AK coordinated the Evoltree project and drafted the manuscript. All authors read and approved the final manuscript.

## Supplementary Material

Additional file 1 **Summary of available BAC libraries in forest tree species**. Click here for file

Additional file 2 **Screening of the *Quercus robur *BAC library with SSR markers**. The file contains the number of amplification products obtained after PCR screening of the oak BAC library (7×) with SSR markers chosen along the 12 linkage group of the oak map, to assess the genome coverage of the BAC library. Click here for file

Additional file 3 **Sequences of the 41 oak- repeat elements (ORE) identified in the BESs dataset**. Click here for file

Additional file 4 **Characteristics of the 41 OREs identified in oak BESs**. The file contains frequencies of OREs and homology searches results against oak sequences available in database (oak contigV1 and NR nucleic database release 3.10-11) Click here for file

Additional file 5 **Frequencies of simple sequence repeats (SSR) in BESs from several plant species**. Click here for file

Additional file 6 **Sequences of the 1,823 oak BESs with a match in the Swissprot database (release 2010-04)**. Click here for file

Additional file 7 **Gene content of the oak BESs**. The file contains homology searches of masked BESs with protein databases: A. thaliana section of the non redundant protein data base (release 03-10-2010), A. thaliana section of the Swissprot database (release 2010-04) and the oak EST database (Oak Contig V1). Click here for file

Additional file 8 **Functional annotation of 1,053 oak BESs significantly aligned with *A. thaliana *sequences in Swissprot**. The table shows the GO terms associated with the coding regions identified on the BESs, annotated with B2GO. Click here for file

Additional file 9 **Comparative genome mapping of the oak BESs with *Vitis vinifera***. BESs were compared with *V. vinifera *genome using the cutoff at 1e-4, with a minimum identity of 90%. Click here for file

Additional file 10 **Comparative genome mapping of the oak BESs with *Populus trichocarpa***. BESs were compared with *P. trichocarpa *genome using the cutoff at 1e-4, with a minimum identity of 90%. Click here for file
