# Supplementary material for: Analysis of BAC end sequences in oak, a keystone forest tree species, providing insight into the composition of its genome
Source: BMC Genomics. 2011 Jun 6;12:292. doi: 10.1186/1471-2164-12-292 (PMC3132169; doi:10.1186/1471-2164-12-292)
Supplement: Additional file 2 — Screening of the Quercus robur BAC library with SSR markers. The file contains the number of amplification products obtained after PCR screening of the oak BAC library (7×) with SSR markers chosen along the 12 linkage group of the oak map, to assess the genome coverage of the BAC library. [file 1471-2164-12-292-S2.PDF]

Screening of the *Quercus robur* BAC library with SSR markers selected along the 12 linkage groups.

Linkage group	Locus name	Number of hits/marker
1	PIE238	3
1	FIR073	4
1	POR054	14
1	ZQR74	11
1	WAG011	4
2	PIE271	6
2	PIE258	3
2	PIE152	10
2	ZQR87	6
2	PIE152	12
3	PIE163	5
3	PIE145	0
3	PIE138	4
3	FIR075	17
3	PIE267	7
4	PIE216	3
4	PIE176	4
4	POR	12
4	CQM4	0
4	PIE228	12
5	PIE246	5
5	ZQR39	5
5	ZQR58	4
5	PIE013	12
5	POR014	8
6	PIE188	3
6	PIE260	20
6	CQM13	6
6	PIE125	13
6	PIE035	9
7	PIE167	7
7	PIE219	6
7	FIR095	8

7	ZQP9	13
7	PIE164	14
8	ZQP110	7
8	ZQR5	3
8	PIE101	0
8	PIE175	4
8	PIE149	1
9	PIE264	8
9	ZQR15	7
9	ZQR31	7
9	SQS22	2
9	PIE059	7
10	PIE041	4
10	PIE148	1
10	ZQR65	10
10	PIE186	10
10	PIE244	8
11	PIE033	14
11	PIE036	2
11	PIE144	6
11	FIR104	4
11	PIE102	1
12	PIE236	4
12	PIE039	9
12	FIR059	9
12	ZQR112	7
12	PIE126	6
