# Supplementary material for: Analysis of BAC end sequences in oak, a keystone forest tree species, providing insight into the composition of its genome
Source: BMC Genomics. 2011 Jun 6;12:292. doi: 10.1186/1471-2164-12-292 (PMC3132169; doi:10.1186/1471-2164-12-292)
Supplement: Additional file 5 — Frequencies of simple sequence repeats (SSR) in BESs from several plant species. [file 1471-2164-12-292-S5.PDF]

species	genome size (Mb)	Number of BESs	BESs total size (Mb)	proportion of genome(%)	SSR number	SSR number per 100Kb	Reference
Quercus robur (Bes)	740	20056	12	0.02	3531	29.43	this article
Citrus clementina (Bes)	400	46000	28.6	0.08	4762	16.65	Terol J. *et al.* 2008 (1)
Carica papaya (Bes)	372	50661	17	4.7	4426	26.04	Lai CW. *et al.* 2006 (2)
Populus trichocarpa (Bes)	370	13249	10.8	0.03	2040	18.89	kindly provided by P. Faivre-Rampant
Brassica oleracea (Bes)	760	85416	67	0.09	7454	11.13	http://brassica.bbsrc.ac.uk/
Glycine max (Bes)	1103	325952	223.8	0.2	32793	14.65	Saini N. *et al.* 2008 (3)
Solanum lycopersicum (Bes)	950	399114	344.4	0.36	24626	7.15	Datema E. *et al.* 2008 (4)
Brassica rapa (Bes)	784	198490	154.6	0.2	16168	10.46	Chang Pyo Hong. *et al.* 2006 (5)
Solanum tuberosum (Bes)	840	140540	91.8	0.11	10617	11.57	Datema E. *et al.* 2008 (4)
Cucumis sativus (Bes)	882	63935	47.6	0.05	6841	14.37	Woycicki,R.*et al.* 2009 (6)
Prunus persica (Bes)	270	47311	37.7	0.14	8479	22.49	Georgi LL. *et al.* 2003 (7)

(1) Terol J, Naranjo MA, Ollitrault P, Talon M.Development of genomic resources for Citrus clementina: characterization of three deep-coverage BAC libraries and analysis of 46,000 BAC end sequences.BMC Genomics. 2008 Sep 18;9:423.

(2) Lai CW, Yu Q, Hou S, Skelton RL, Jones MR, Lewis KL, Murray J, Eustice M, Guan P, Agbayani R, Moore PH, Ming R, Presting GG. Analysis of papaya BAC end sequences reveals first insights into the organization of a fruit tree genome. Mol Genet Genomics. 2006 Jul;276(1):1-12. Epub 2006 May 16.

(3) Saini N, Shultz J, Lightfoot DA. Re-annotation of the physical map of Glycine max for polyploid-like regions by BAC end sequence driven whole genome shotgun read assembly. BMC Genomics. 2008 Jul 7;9:323.

(4) Datema E, Mueller LA, Buels R, Giovannoni JJ, Visser RG, Stiekema WJ, van Ham RC. Comparative BAC end sequence analysis of tomato and potato reveals overrepresentation of specific gene families in potato. BMC Plant Biol.

(5) Chang Pyo Hong, Prikshit Plaha, Dal-Hoe Koo, Tae-Jin Yang, Su Ryun Choi, Young Ki Lee, Taesik Uhm, Jae-Wook Bang, David Edwards, Ian Bancroft, Beom-Seok Park, Jungho Lee, and Yong Pyo Lim. A Survey of the Brassica rapa Genome 2008 Apr 11;8:34. by BAC-End Sequence; Analysis and Comparison with Arabidopsis thaliana. Mol. Cells, Vol. 22, No. 3, pp. 300-307

(6) Woycicki,R., Malepszy,S., Plader,W., Witkowicz,J., Przybecki ,Z. Cloning of the sex genes of cucumber (Cucumis sativus L.): Sequencing of the BAC library ends (2009). Unpublished

(7) Georgi LL, Wang Y, Reighard GL, Mao L, Wing RA, Abbott AG. Comparison of peach and Arabidopsis genomic sequences: fragmentary conservation of gene neighborhoods. Genome. 2003 Apr;46(2):268-76.
