# Supplementary material for: Analysis of BAC end sequences in oak, a keystone forest tree species, providing insight into the composition of its genome
Source: BMC Genomics. 2011 Jun 6;12:292. doi: 10.1186/1471-2164-12-292 (PMC3132169; doi:10.1186/1471-2164-12-292)
Supplement: Additional file 7 — Gene content of the oak BESs. The file contains homology searches of masked BESs with protein databases: A. thaliana section of the non redundant protein data base (release 03-10-2010), A. thaliana section of the Swissprot database (release 2010-04) and the oak EST database (Oak Contig V1). [file 1471-2164-12-292-S7.PDF]

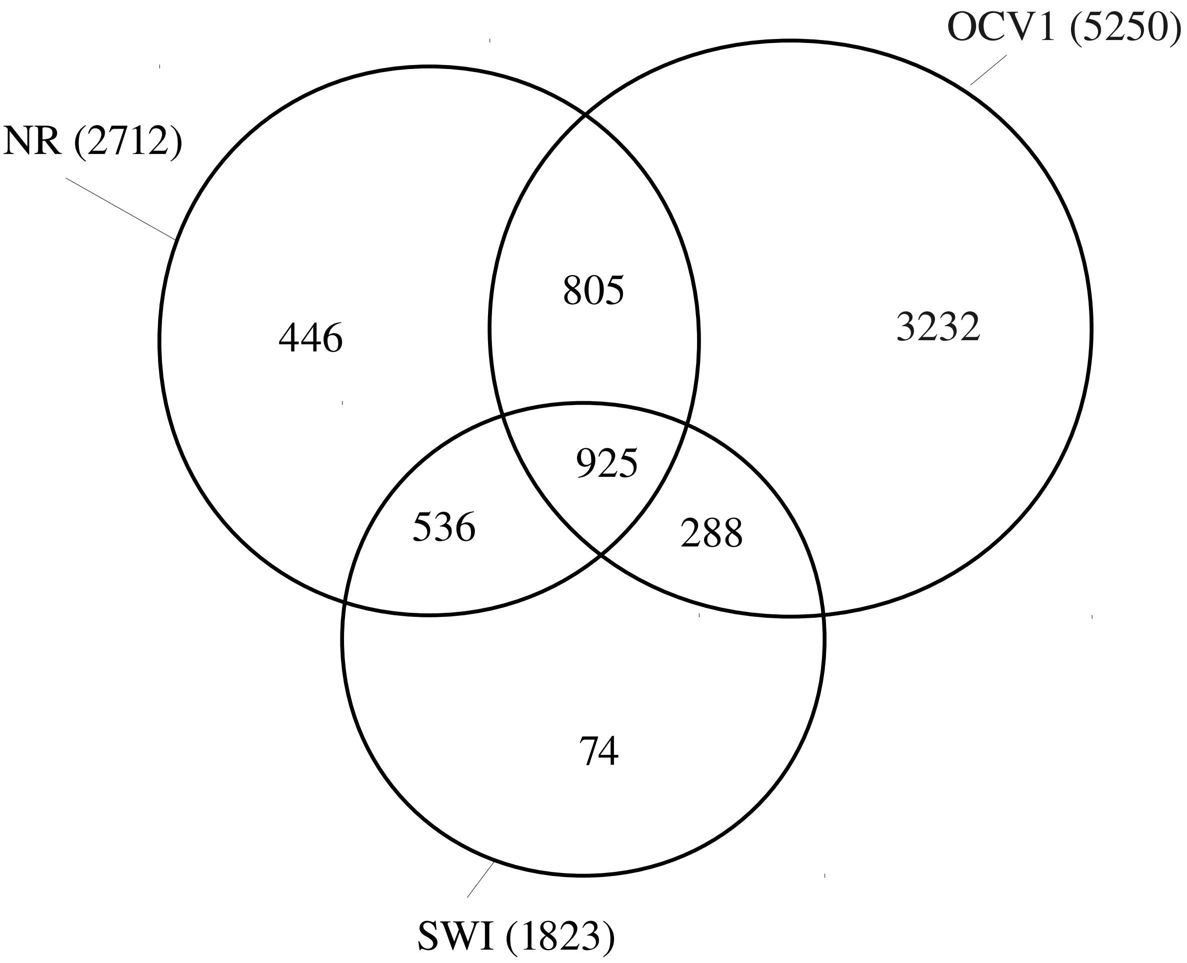

Results of sequence homology searches of masked BESs with protein databases (*A. thaliana* section of the non-redundant protein database – red circle, *A. thaliana* section of the Swissprot database – blue circle) and EST databases (Oak Contig V1 EST database – black circle) are represented by a Venn diagram. Numbers correspond to the number of BESs which matched at least one sequence in the target database. Among the 5,250 BESs identified by similarity search in the OCV1 database, 2,018 (38.44%) also matched at least one sequence in either Swissprot, or NR or both databases.
